# Ectopic Expression of *JcCPL1*, *2*, and *4* Affects Epidermal Cell Differentiation, Anthocyanin Biosynthesis and Leaf Senescence in *Arabidopsis thaliana*

**DOI:** 10.3390/ijms23041924

**Published:** 2022-02-09

**Authors:** Yanbo Chen, Pingzhi Wu, Chao Zhang, Yali Guo, Bingbing Liao, Yaping Chen, Meiru Li, Guojiang Wu, Yaqin Wang, Huawu Jiang

**Affiliations:** 1 Guangdong Provincial Key Laboratory of Biotechnology for Plant Development, School of Life Sciences, South China Normal University, Guangzhou 510631, China; chenyanbo1221@163.com (Y.C.); liao_bbing@163.com (B.L.); 2 Key Laboratory of Plant Resources Conservation and Sustainable Utilization, South China Botanical Garden, Chinese Academy of Sciences, Guangzhou 510650, China; pzwu@scbg.ac.cn (P.W.); guoyali1129@126.com (Y.G.); chenyp@scbg.ac.cn (Y.C.); limr@scbg.ac.cn (M.L.); wugj@scbg.ac.cn (G.W.); 3 Key Laboratory of South Subtropical Fruit Biology and Genetic Resource Utilization, Ministry of Agriculture/Key Laboratory of Tropical and Subtropical Fruit Tree Research of Guangdong Province, Institution of Fruit Tree Research, Guangdong Academy of Agricultural Sciences, Guangzhou 510640, China; 4 College of Agronomy, Northwest A&F University, Xianyang 712100, China; ahzc2009@163.com

**Keywords:** MYB-related transcription factor, physic nut (*Jatropha curcas* L.), epidermal cell differentiation, anthocyanin, leaf senescence

## Abstract

The CAPRICE (CPC)-like (CPL) genes belong to a single-repeat R3 MYB family, whose roles in physic nut (*Jatropha curcas* L.), an important energy plant, remain unclear. In this study, we identified a total of six CPL genes (*JcCPL1*–*6*) in physic nut. The JcCPL3, 4, and 6 proteins were localized mainly in the nucleus, while proteins JcCPL1, 2, and 5 were localized in both the nucleus and the cytoplasm. Ectopic overexpression of *JcCPL1*, *2*, and *4* in *Arabidopsis thaliana* resulted in an increase in root hair number and decrease in trichome number. Consistent with the phenotype of reduced anthocyanin in shoots, the expression levels of anthocyanin biosynthesis genes were down-regulated in the shoots of these three transgenic *A. thaliana* lines. Moreover, we observed that *OeJcCPL1*, *2*, *4* plants attained earlier leaf senescence, especially at the late developmental stage. Consistent with this, the expression levels of several senescence-associated and photosynthesis-related genes were, respectively, up-regulated and down-regulated in leaves. Taken together, our results indicate functional divergence of the six CPL proteins in physic nut. These findings also provide insight into the underlying roles of CPL transcription factors in leaf senescence.

## 1. Introduction

MYB transcription factors constitute one of the largest families of plant transcription factors. A protein encoded by an MYB gene usually includes one to four incompletely repeated MYB domains, which are named R1, R2 and R3 according to their sequence similarity with the c-MYB protein [1,2]. A MYB domain generally contains 52 amino acid residues which form three a-helices, of which the second and third make up a structure that interacts with the major groove of DNA [1,2]. According to the number of MYB domains, the MYB transcription factor superfamily can be divided into four families: R2R3-MYB, 3R-MYB, 4R-MYB and MYB-related [3]. MYB-related proteins usually contain a single MYB domain, but sometimes there are two MYB domains, and they have been classified into five subfamilies: CCA1-like, CAPRICE (CPC)-like (CPL), TBP-like, I-box-binding-like and R-R-type [4].

The CPL proteins contain a single R3 MYB domain [5]. There is a total of seven CPL genes in the *Arabidopsis thaliana* genome: *ETC1* (At1g01380), *TCL1* (At2g30432), *TCL2* (AT2G30424), *ETC2* (At2g30420), *CPC* (At2g46410), *ETC3*/*CPL3* (At4g01060) and *TRY* (At5g53200) [4]. They are involved in anthocyanin synthesis, differentiation of trichomes and root hair development [5,6,7,8,9,10,11,12]. These characters have been reported to be governed by the MYB-bHLH-WD40 (MBW) protein complex, which consists of R2R3-MYB factors, a bHLH transcription factor and a WD40 repeat domain containing protein [13,14,15,16,17,18]. CPL transcription factors can compete with the R2R3-MYB transcription factor, thus inhibiting the formation of the MBW complex [19]. In the root, the MBW protein complex, which is composed of WEREWOLF (WER), GLABRA3/ENHANCER OF GLABRA3 (GL3/EGL3) and TRANSPARENT TESTA GLABRA1 (TTG1), inhibits the formation of root hairs by directly activating the expression of *GLABRA2* (*GL2*) [5,20,21,22,23,24]. In shoots, the MBW complex, which is made up of GLABRA1 (GL1), GL3/EGL3 and TTG1, promotes the expression of *GL2*, whose function is completely opposite in root and shoot, and thus promotes trichome initiation [25,26,27,28,29]. CPL transcription factors, which can move between non-hair cells and adjacent cells, compete with GL1/WER, and prevent the formation of GL1/WER-GL3/EGL3-TTG1 complex, then inhibit the expression of the *GL2* gene, thereby promoting the differentiation of root hairs and inhibiting trichome initiation [8,27,28,30,31,32,33,34].

Moreover, CPL transcription factors compete with the R2R3-MYB transcription factor (PAP1/2), preventing the formation of MBW protein complexes, and negatively regulating anthocyanin biosynthesis, which can be divided into early pathways and late pathways [15,16,19,35]. Early biosynthesis genes (EBGs) of anthocyanin include chalcone synthase (CHS), chalcone isomerase (CHI), and flavanone 3-hydroxylase (F3H), which are regulated mainly by three redundant R2R3-MYB transcription factors, MYB11/12/111 [35,36,37,38]. Late biosynthesis genes (LBGs) of anthocyanin, including dihydroflavonol 4-reductase (DFR), anthocyanidin synthase (ANS) and UDP-glucose flavonoid glucosyltransferase (UFGT), are positively regulated by the MBW protein complex [15,35,38,39]. Overexpressing *AtCPC* in *A. thaliana* represses anthocyanin accumulation under nitrogen deficiency conditions, and the expression of LBGs including *DFR* and *ANS* also significantly decreases; the opposite trend is shown in the *cpc*-*1* mutant [11]. Similarly, ectopic expression of *AtCPC* in tobacco efficiently decreases the anthocyanin content of flowers, as well as the expression of LBGs (*NtDFR*, *Nt3GT* and *NtANS*) [40].

Root hairs are single cell tubular outgrowths of root epidermal cells, which enlarge the surface area of roots and are often thought to help plants obtain water and nutrients [41]. Understanding the occurrence, development and genetic pattern of root hairs can provide a favorable basis for the study of nutrient uptake in plants. Trichomes, another type of specialized epidermal cells, are found on the epidermis of most land plants, and their presence can increase the thickness of the protective layer of plant epidermal tissue, reduce heat and water loss of plant, and involve in plant resistance against herbivores [42,43]. Anthocyanins are an important antioxidant due to their higher antioxidant activity than other flavonoids [44]. Moreover, anthocyanins are important members of the color of the appearance of flowers. In particular, they have applications in ornamental plants to regulate flower color, such as the production of pure white petals by downregulation of anthocyanin. Therefore, the study of anthocyanin biosynthetic pathways is of particular importance.

Physic nut (*Jatropha curcas* L.), a tropical shrub, is an important biofuel crop, which is well adapted to barren land [45,46,47]. Our previous study identified MYB and MYB-like family members in the physic nut genome [48,49]. In this study, we characterized the exon-intron organization and conserved domains of the six physic nut CPL genes, and analyzed their spatial and temporal expression patterns. Ectopic overexpression of three (*JcCPL1*, *2*, *4*) of the six CPL genes in *A. thaliana* revealed their roles in epidermal development and anthocyanin biosynthesis, and also provided insight into their underlying roles in leaf senescence.

## 2. Results

### 2.1. Identification and Phylogenetic Analysis of CPL Transcription Factors in Physic Nut

**Figure 1:**
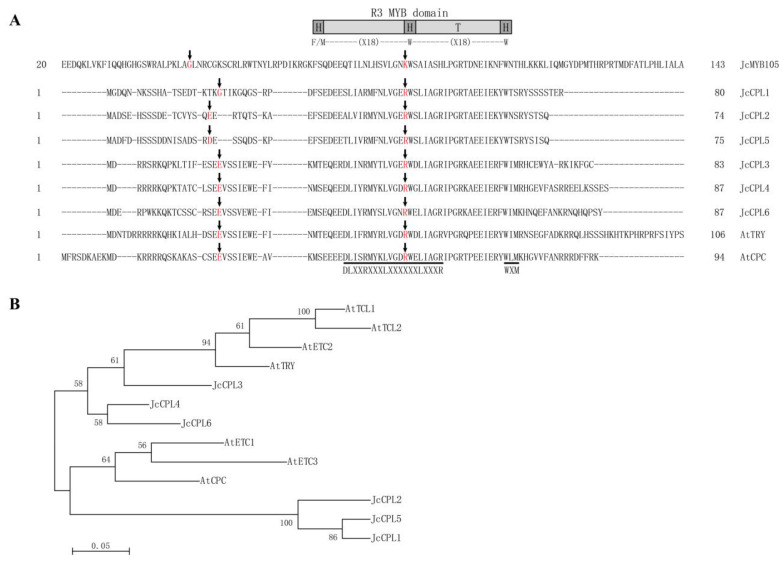
Amino acid sequence alignment and phylogenetic analysis. (**A**) Alignment of the amino acids of the R2 and R3 repeats of representative R2R3-MYB (JcMYB105) and CPL proteins, indicating the exon-intron structure models. Intron positions, relative to the amino acid residues, which are marked in red, are indicated by arrows. Arrows pointing to amino acids indicate that splicing occurred within the amino acid coding sequences. The sequences of two conserved amino acids motifs are marked with black lines. The primary and secondary structures of a typical R3 MYB according to Dubos et al. (2010) [3] are indicated. H, helix; T, turn; F, Phenylalanine; M, Methionine, W, Tryptophan; X, any amino acid. (**B**) Phylogenetic analysis of the CPC-like subfamily of *A. thaliana* and physic nut using amino acid sequences. Bootstrap values from 100 replications are indicated at the branch points. The tree is drawn to scale, with branch lengths in the same units as those of the evolutionary distances that were used to infer the phylogenetic tree.

To identify CAPRICE (CPC)-like (CPL) genes in physic nut, BLAST searches were carried out against the public physic nut genome and proteome database [48,50], using the amino acid sequences of *Arabidopsis thaliana* CPC proteins as queries. As a result, a total of six loci putatively encoding CPLs were identified and named *JcCPL1* to *JcCPL6*; they are located on chromosome 3 (*JcCPL1* and *JcCPL2*), 5 (*JcCPL3*), 8 (*JcCPL4*), 7 (*JcCPL5*), and 9 (*JcCPL6*). All *JcCPL* genes consist of three exons and two introns, and the proteins they encode contain 74 to 87 amino acid residues. After analyzing the overall exon-intron arrangements, we observed that *JcCPL* genes share the same structure as model Ia and Ic R3 type MYB domains [49], which have an intron within the MYB domain coding sequences (Figure 1A).

To examine the evolutionary relationships of physic nut CPL genes, a phylogenetic analysis was carried out using CPL proteins from physic nut and *A. thaliana*. The results indicate that CPL proteins were divided into two groups: JcCPL3, JcCPL4 and JcCPL6 were more closely related to the TRY group (group I) of *A. thaliana*, while JcCPL1, JcCPL2 and JcCPL5 were more closely related to the CPC group (group II) of *A. thaliana* (Figure 1B).

The JcCPL proteins have a conserved R3 MYB domain, but variable N-terminal and C-terminal regions. According to a previous study, the conserved amino acid motif (Motif 1, M1) of [D/E]LX_2_[R/K]X_3_LX_6_LX_3_R (X represents any amino acid), located on helix 1 and helix 2 of the R3 MYB domain, is crucial for interaction between R3 MYB and bHLH proteins [14]. Another motif, WXM (Motif 2, M2), which is located on helix 3 of the MYB domain, is related to cell-to-cell movement of the CPC protein in *A. thaliana* [31]. Our results show that the two amino acid motifs in group I JcCPL proteins (JcCPL3, JcCPL4 and JcCPL6) were conserved. However, in group II JcCPL proteins (JcCPL1, JcCPL2 and JcCPL5), the first amino acid D/E in the M1 motif was replaced by S, A or T, and the amino acid M in the M2 motif was replaced by S (Figure 1A).

### 2.2. Expression of JcCPL Genes in Different Organs

**Figure 2:**
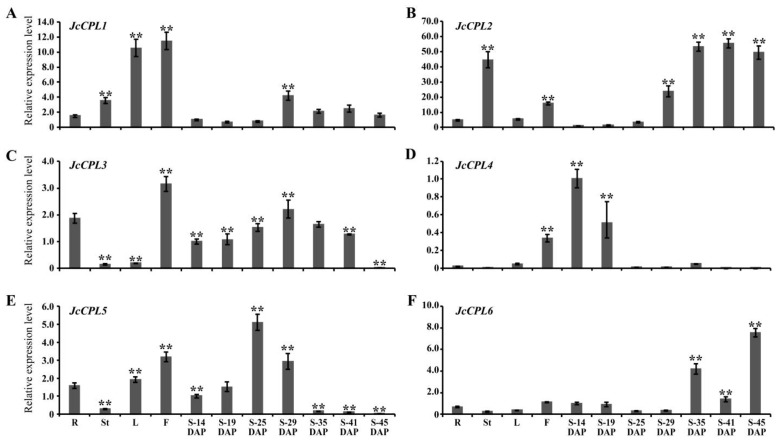
Expression patterns of JcCPL genes. (**A**–**F**) The relative expression levels of *JcCPL1* (**A**), *JcCPL2* (**B**), *JcCPL3* (**C**), *JcCPL4* (**D**), *JcCPL5* (**E**) and *JcCPL6* (**F**) in different organs were tested by qRT-PCR. Relative expression was normalized to the reference gene *JcActin* (GenBank accession number HM044307.1) and the expression level at S-14 DAP was defined as “1”. R, roots; St, young bark of stem; L, leaf; F, flower; S-14–45 DAP, developing seeds at 14–45 days after pollination. Values are means ± SD (Duncan test: ** *p* < 0.01) of three biological replicates and three technical replicates.

After detecting ESTs representing six *JcCPL* genes by analysing the expressed sequence tag (EST) databases of physic nut [48], we measured the expression levels of six *JcCPL* transcripts by qRT-PCR using leaves, roots, and young bark of stems from 8-week-old plants, and flowers and developing seeds from 5-year-old plants. The results show that the *JcCPL1* gene was highly expressed in flowers and leaves (Figure 2A). *JcCPL2* was expressed highly in young bark and in the middle and late developmental stages of seeds (Figure 2B). The *JcCPL3* gene was expressed at high levels in roots, flowers and in the early and middle developmental stages of seeds (Figure 2C). *JcCPL4* was expressed highly in flowers and in the early developmental stage of seeds (Figure 2D). *JcCPL5* was expressed highly in roots, leaves, flowers and in the early developmental stage of seeds (Figure 2E), while *JcCPL6* was expressed mainly in seeds in the middle and late developmental periods (Figure 2F). The different expression patterns of *JcCPLs* in different organs of physic nut implied that they may possess functional diversity.

### 2.3. Subcellular Localization of JcCPL Proteins

**Figure 3:**
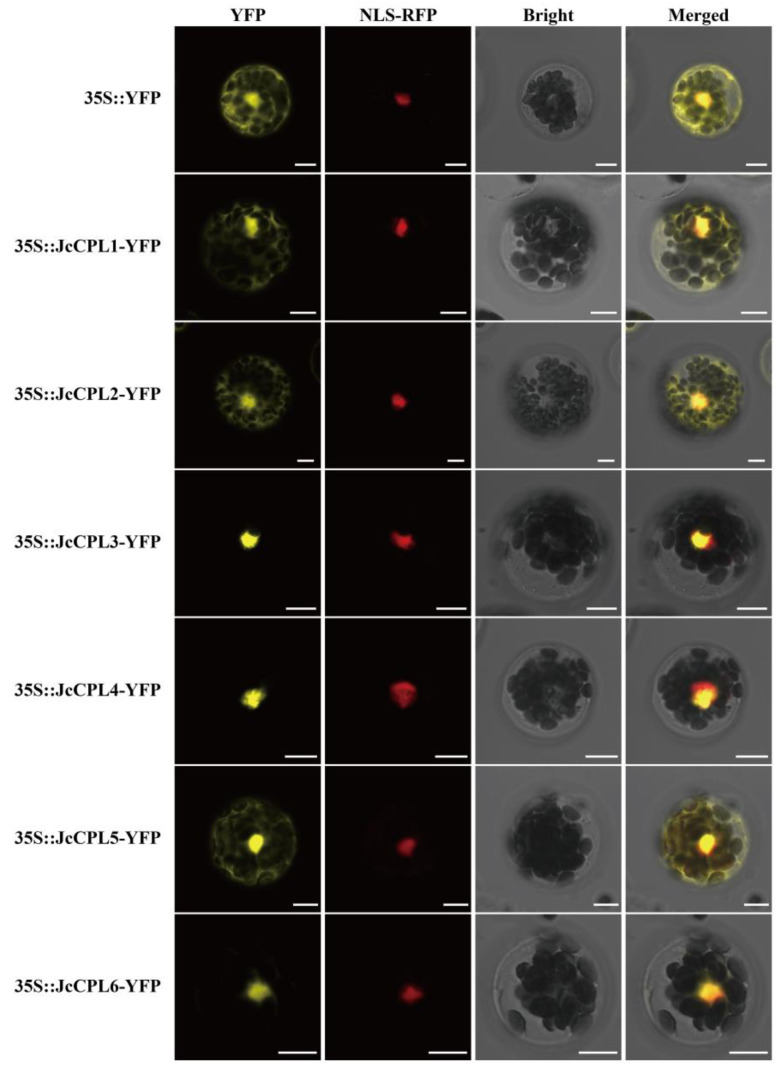
Subcellular localization of JcCPL proteins in *A. thaliana* leaf protoplasts. A construct containing the naked *YFP* gene was used as the control. The constructs were co-transformed into *A. thaliana* protoplasts with NLS-RFP. Scale bar = 10 μm.

To determine the subcellular localizations of JcCPL proteins, we made constructs each consisting of the coding domain sequence of one of the *JcCPL* genes fused with a *YFP* gene under the control of the cauliflower mosaic virus (CaMV) 35S promoter in a transient expression vector. Each recombinant was transformed into *A. thaliana* protoplasts. For JcCPL1, JcCPL2 and JcCPL5 proteins, the fluorescent signals were detected in both cell nuclei and cytoplasm, whilst signals for JcCPL3, JcCPL4 and JcCPL6 were mainly in cell nuclei (Figure 3).

### 2.4. Overexpression of JcCPL1, 2, and 4 Influences the Formation of Trichomes and Root Hairs in A. thaliana

**Figure 4:**
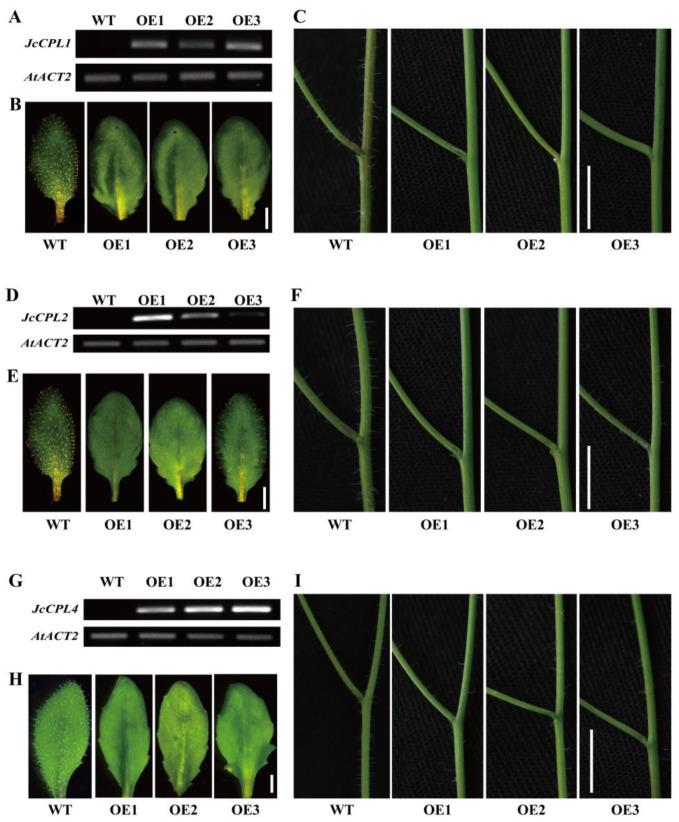
Overexpression of *JcCPL1*, *JcCPL2* and *JcCPL4* modulates trichome development in *A. thaliana* under normal conditions. (**A**,**D**,**G**) Expression levels of *JcCPL1* (**A**), *JcCPL2* (D) and *JcCPL4* (**G**) in transgenic lines (OE1, OE2 and OE3) determined by semi-quantitative RT-PCR. (**B**,**E**,**H**) Leaf trichome phenotypes in *OeJcCPL1* (**B**), *OeJcCPL2* (**E**) and *OeJcCPL4* (**H**) maintained under normal conditions for 28 days. Scale bar = 2 mm. (**C**,**F**,**I**) Phenotypes of inflorescence stem trichomes in *OeJcCPL1* (**C**), *OeJcCPL2* (**F**) and *OeJcCPL4* (**I**) grown under normal conditions for 35 days. Photos were taken of the first branch of the main inflorescence stem within 15 cm from the base. Scale bar = 5 mm.

To investigate the biological roles of *JcCPL* genes, each of these genes was overexpressed in *A. thaliana* and three homozygous transgenic *A. thaliana* lines for each gene were obtained. Semi-quantitative RT-PCR showed that the expression levels of the six *JcCPL* genes increased to different degrees in the transgenic lines (Figure 4A,D,G; Supplementary Figure S1A,D,G). However, there were no significant differences in plant size between overexpressing plants and wild type *A. thaliana* (WT) (Supplementary Figure S1B,E,H; Supplementary Figure S2).

**Figure 5:**
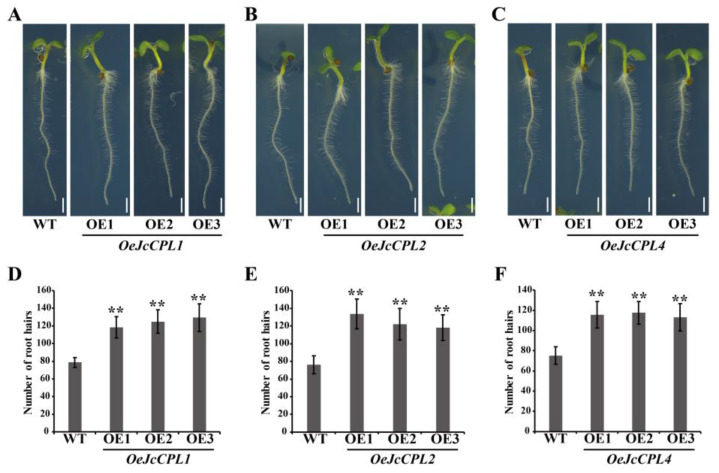
Phenotypes and root hair numbers of *OeJcCPL1*, *OeJcCPL2* and *OeJcCPL4* seedlings. (**A**–**C**) Seedlings grown in 1/2 MS for 4 days: *OeJcCPL1* (**A**), *OeJcCPL2* (**B**) and *OeJcCPL4* (**C**); scale bar = 1 mm. (**D**–**F**) Root hair numbers of *OeJcCPL1* (**D**), *OeJcCPL2* (**E**) and *OeJcCPL4* (**F**). Error bars were calculated from the means ± SD (Duncan test: ** *p* < 0.01) of a minimum of twenty seedlings from each line.

*A. thaliana* R3 MYB genes are involved in trichome formation and root hair formation [51]. In this study, we found that overexpression of *JcCPL1*, *JcCPL2*, or *JcCPL4* in *A. thaliana* (*OeJcCPL1*, *2*, *4*) resulted in a major reduction in trichome formation on rosette leaves and inflorescence stems (Figure 4B,C,E,F,H,I). Line 3 (OE3) of *OeJcCPL2* formed some trichomes on its leaves and inflorescence stems, probably due to the low expression level in this line. In addition, the number of root hairs increased significantly in *OeJcCPL1*, *2*, and *4* plants compared with WT plants (Figure 5). Unexpectedly, no significant changes were observed in the root hairs of overexpression *JcCPL3*, *JcCPL5*, or *JcCPL6* plants (*OeJcCPL3*, *5*, *6*) (Supplementary Figure S1C,F,I). Similarly, no abnormal trichome formation was observed on leaves and inflorescence stems in *OeJcCPL3*, *5* and *6* (data not shown). Accordingly, these three genes were not studied in subsequent experiments.

### 2.5. Overexpression of JcCPL1, 2, and 4 Reduces Anthocyanin Content in A. thaliana

**Figure 6:**
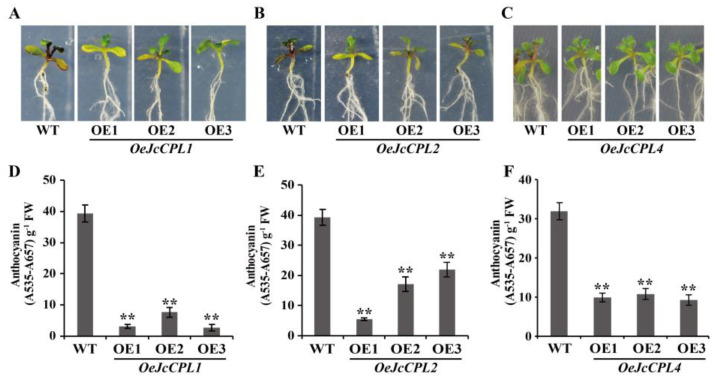
Phenotypes and anthocyanin contents of *OeJcCPL1*, *OeJcCPL2* and *OeJcCPL4* seedlings. (**A**–**C**) Shoot phenotypes of *OeJcCPL1* (A), *OeJcCPL2* (**B**) and *OeJcCPL4* (**C**) seedlings. The seedlings were germinated on 1/2 MS with 1% sucrose for 4 days and then transferred to anthocyanin induction medium (AIM) containing 5% sucrose for 7 days. (**D**–**F**) Anthocyanin contents in shoots of *OeJcCPL1* (**D**), *OeJcCPL2* (**E**), and *OeJcCPL4* (**F**) plants. Values are means of *n* = 3 ± SD (Duncan test: ** *p* < 0.01).

The CPL genes also play roles in regulating anthocyanin biosynthesis in plants [40,52] and CPC is a negative regulator of anthocyanin biosynthesis [11]. The anthocyanin content of *A. thaliana* shoots was very low under normal growth condition, so the content was measured in plants grown on an anthocyanin-inducing medium (AIM). In this study, the sterilized seeds were sown in 1/2 MS medium for 4 days, and then transferred to AIM containing 5% sucrose for 7 days. Subsequently, a lower level of anthocyanin accumulation in shoots could be easily observed in *OeJcCPL1*, *2*, and *4* shoots (Figure 6A–C). In most shoots of *OeJcCPL1*, *2*, and *4* plants, the anthocyanin contents were 50% lower than in WT (Figure 6D–F).

**Figure 7:**
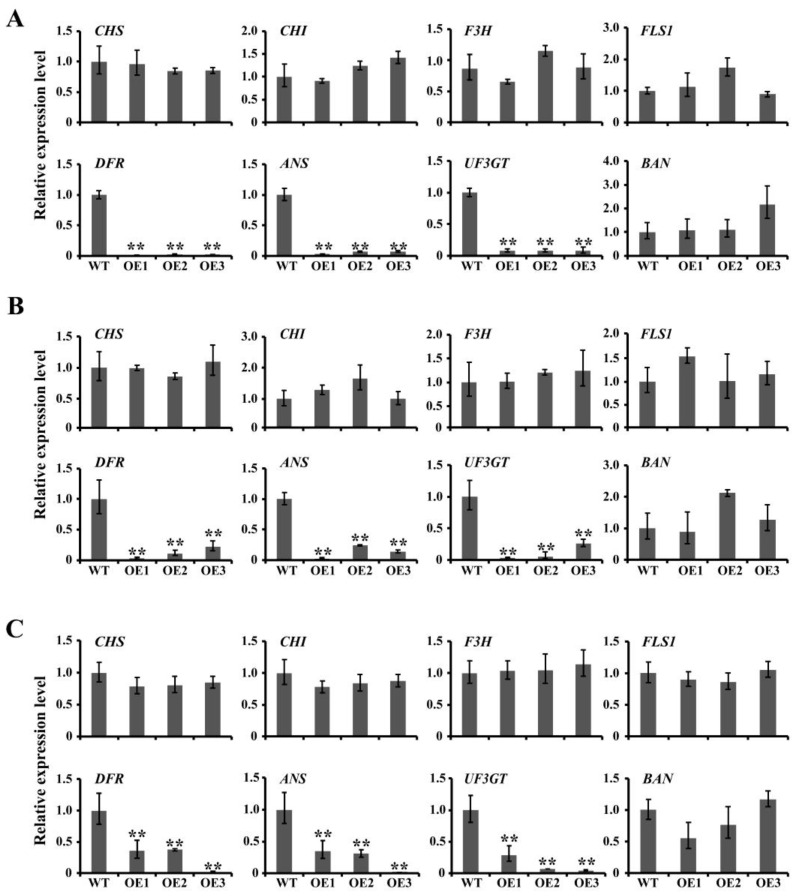
Expression analysis of anthocyanin biosynthesis-related genes by qRT-PCR. (**A**) *OeJcCPL1* lines. (**B**) *OeJcCPL2* lines. (**C**) *OeJcCPL4* lines. Seeds were germinated on 1/2 MS medium for 4 days, and 1/2 MS medium containing 5% sucrose for 7 days. Whole shoots were sampled for the qRT-PCR analysis. *CHS* (chalcone synthase, At5g13930); *CHI* (chalcone isomerase, At3g55120); *F3H* (flavanone 3-hydroxylase, At3g51240); *FLS1* (flavonol synthase 1, AT5G08640); *DFR* (dihydroflavonol 4-reductase, AT5G42800); *ANS* (anthocyanidin synthase, AT4G22880); *UF3GT* (UDP-glucose: flavonoid 3-O-glucosyltransferase, AT5G54060); *BAN* (anthocyanidin reductase, AT1G61720). *AtACT2* (At3g18780) was used as an internal control. Values are means of *n* = 3 ± SD (Duncan test: ** *p* < 0.01).

The anthocyanin biosynthesis pathway can be divided into early biosynthesis genes (EBGs) (including *CHS*, *CHI* and *F3H*) and late biosynthesis genes (LBGs) (including *DFR*, *ANS*, *UFGT*) [15,35,36]. The *FLS1* and *BAN* genes are involved in the biosynthesis of flavonoids and proanthocyanidins, respectively [15,38]. To determine which steps were down-regulated in the anthocyanin biosynthesis pathway in *JcCPL1*, *JcCPL2* and *JcCPL4* overexpressing plants, the expression levels of several relevant genes were tested by qRT-PCR analysis. The results show that the expression levels of *CHI*, *CHS*, *F3H*, *FLS1* and *BAN* gene in *OeJcCPL1*, *OeJcCPL2* and *OeJcCPL4* shoots were not significantly changed when compared with WT shoots (Figure 7). However, the expression levels of *DFR*, *ANS* and *UF3GT* were significantly lower in these shoots than in WT (Figure 7). These results indicated down-regulation of the LBGs of anthocyanin synthesis pathway in the *OeJcCPL1*, *OeJcCPL2* and *OeJcCPL4* shoots.

### 2.6. Overexpression of JcCPL1, 2, and 4 Promotes Leaf Senescence in A. thaliana

**Figure 8:**
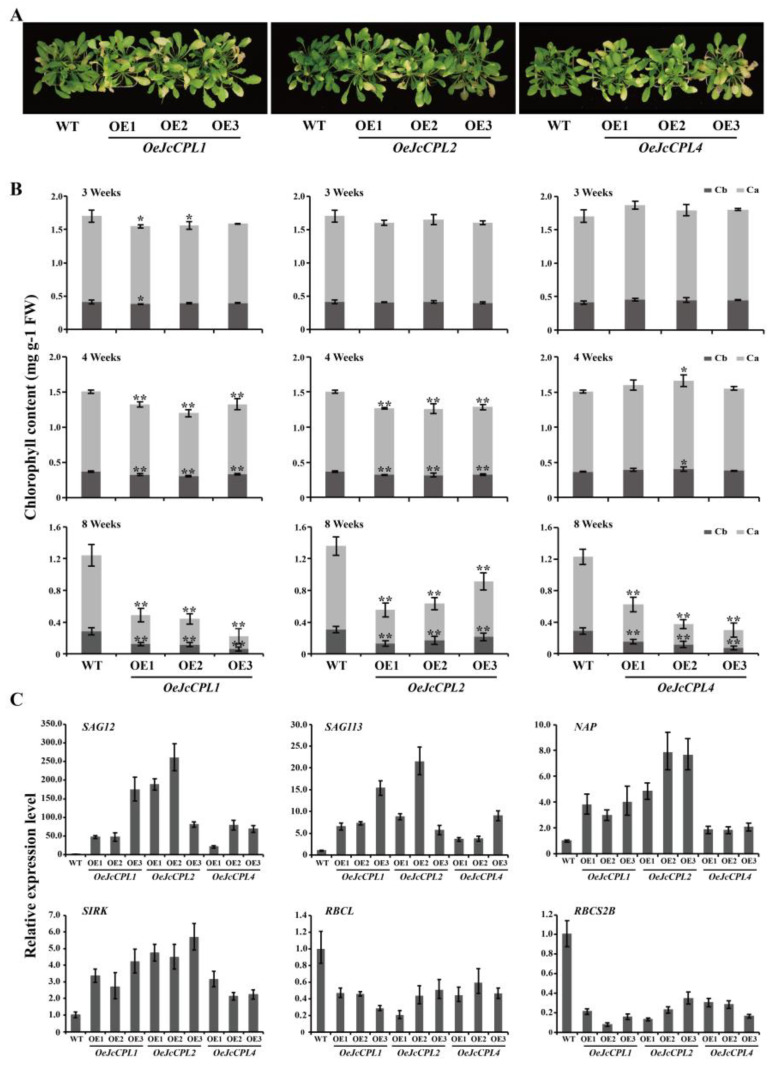
Chlorophyll contents and expression analysis of senescence-related and photosynthesis-related genes in leaves of *OeJcCPL1*, *OeJcCPL2* and *OeJcCPL4* lines. (**A**) Eight-week-old plants of WT and *OeJcCPL1*, *OeJcCPL2* and *OeJcCPL4* lines with inflorescences removed. (**B**) Chlorophyll contents of *OeJcCPL1*, *OeJcCPL2* and *OeJcCPL4* lines at 3 weeks, 4 weeks and 8 weeks. (**C**) Expression analysis of senescence-related and photosynthesis-related genes. *SAG12* (senescence-associated gene 12, At5g45890); *SAG113* (senescence-associated gene 113, At5g59220); *NAP* (Arabidopsis NAC domain containing protein, At1g69490); *SIKR* (senescence-induced receptor-like kinase, AT2G19190); *RBCL* (large subunit of RUBISCO, AtCg00490); *RBCS2B* (RUBISCO small subunit 2B, AT5G38420). *AtACT2* (At3g18780) was used as an internal control. Three replicates were used in the experiment. Values are means of *n* = 3 ± SD (Duncan test: * *p* < 0.05, ** *p* < 0.01).

Leaf chlorophyll content is an important indicator of plant senescence [53]. When monitoring leaf development, we found that the leaves of *OeJcCPL1*, *2*, and *4* transgenic plants turned yellow earlier than those of WT plants. Most rosette leaves of WT appeared green apart from a few lower leaves under routine cultivation conditions. In contrast, most leaves of *OeJcCPL1*, *2*, and *4* transgenic plants appeared yellow with the exception of some upper leaves under the same cultivation conditions (Figure 8A). To further investigate the roles of *JcCPL1*, *2*, and *4* in plant senescence, the chlorophyll contents of leaves were measured at different developmental stages. At the 3-week stage (before flowering), only two lines of *OeJcCPL1* had significantly lower chlorophyll content compared to WT (Figure 8B). At the 4-week stage (after flowering), the chlorophyll content of both *OeJcCPL1* and *OeJcCPL2* plants was significantly decreased compared to WT (Figure 8B). At the 8-week stage (late developmental stage), the chlorophyll contents of *OeJcCPL1*, *2*, and *4* plants were all significantly decreased compared to WT. These results indicate that *JcCPL1*, *2* and *JcCPL4* may promote leaf senescence at different stages of plant growth and development.

To explore the relationship between chlorophyll content and senescence or photosynthesis-related genes, we measured the expression levels of senescence- or photosynthesis-related genes using 8-week-old leaves of *OeJcCPL1*, *2*, and *4* transgenic plants. Consistent with their phenotype, the expression levels of several senescence-related (*SAG12*, *SAG113*, *NAP* and *SIRK*) were increased (Figure 8C), while the expression levels of photosynthesis-related genes (*RBCL* and *RBCS2B*) were decreased, in *OeJcCPL1*, *2*, and *4* leaves compared to those in WT leaves (Figure 8C). These results implied that genes *JcCPL1*, *2*, and *4* play roles in the positive regulation of leaf senescence at different stages of plant development.

## 3. Discussion

In this study, six CAPRICE (CPC)-like (CPL) genes, *JcCPL1*-*JcCPL6*, were identified in the physic nut genome. Analysis of the structure of these revealed that the genes encoding the single repeat R3 MYB region of the six physic nut CPLs have the same exon-intron arrangement and the proteins they encode have high sequence similarity at the amino acid level in the MYB domain region. Our unrooted phylogenetic tree indicated that physic nut CPL proteins fell into two groups: JcCPL3, 4, and 6 (group I), and JcCPL1, 2, and 5 (group II) (Figure 1). This result is consistent with previous phylogenetic analysis which indicated that the seven CPL genes of *Arabidopsis thaliana* were divided into two groups, *AtTCL1*, *AtTCL2*, *AtETC2* and *AtTRY* in group I, and *AtCPC*, *AtETC1* and *AtETC3* in group II [54]. The JcCPL proteins exhibited many divergences at the amino acid level, with variable amino acid sequences at the N- and C- terminals as well as in the conserved motifs within the MYB domain. In the M1 and M2 motifs, several amino acid residues were not conserved in group II JcCPL proteins (Figure 1). These results indicate that divergence of motifs within the JcCPL proteins had occurred before the differentiation of *A. thaliana* and physic nut, especially between group I and group II proteins.

Previous studies indicated that CPL proteins in plants were localized in the nucleus as well as the cytoplasm or plasma membrane [8,55,56,57], or mainly in the nucleus [54]. In this study, we found that group I JcCPL proteins were localized mainly in the nucleus while group II JcCPL proteins were present in both the nucleus and cytoplasm under the same conditions. These results imply that amino acid divergence between the two group JcCPL proteins determined their different subcellular locations. As well as protein subcellular localization, spatiotemporal expression patterns are assumed to reflect the roles of genes [33]; in the present study, the differential patterns of expression indicate that 6 *JcCPL* genes may have different roles in the growth and development of physic nut.

Ectopic expression of the genes *JcCPL1*, *2*, and *4* in *A. thaliana* resulted in increased numbers of root hairs and decreased numbers of trichomes on leaves and inflorescence stems. Anthocyanin accumulation and the expression of late anthocyanin biosynthesis genes were also reduced in the transgenic *A. thaliana* plants. These results are consistent with previous reports on the functions of CPL genes from *A. thaliana* [7,10,58], tomato [52,59] and rice [56]. Thus, these JcCPL proteins may play similar roles to those previously reported for CPL proteins from other plant species. In the root, JcCPL1, 2, and 4 transcription factors might compete with the R2R3-MYB transcription factor (WER), promote the formation of root hairs by inhibiting the formation of the MBW (WER-GL3/EGL3-TTG1) complex [5,8,20,21,22,23,24,27,28,30,31]. In shoots, JcCPL1, 2, and 4 transcription factors might compete with the R2R3-MYB transcription factor (GL1/PAP1/PAP2), inhibit the formation of trichome and anthocyanin accumulation by inhibiting the formation of the MBW (GL1/PAP1/PAP2-GL3/EGL3-TTG1) complex [15,16,19,25,26,27,28,29,33,35]. Meanwhile, whether *JcCPL1*, *2*, and *4* genes regulate epidermal cell differentiation and anthocyanin biosynthesis in physic nut remains to be studied.

However, none of the phenotypes mentioned above were observed in *JcCPL3*-, *5*-, or *6*- overexpressing plants. Similar results have been observed in plants expressing *TRY* and *ETC2*, which did not show any obvious induction of root hairs [60]. Studies have indicated that the TRY and ETC2 members of the CPC family are characterized by rapid proteolysis, which is related to the extended C-terminal domain of these proteins, compared with that of other members [60]. Substitution of amino acids in the C-terminal regions of TRY and ETC2 conferred on them the ability to induce root hair formation [61]. These results indicate that the precise structure of each CPL protein is important in determining its properties. We therefore speculate that this might be one of the reasons why plants overexpressing the *JcCPL3*, *5*, and *6* genes have no visible phenotype. However, further experiments are still needed to explore the specific mechanisms by which the products of *JcCPL3*, *5*, and *6* act.

In addition, we found that the chlorophyll content of both *OeJcCPL1* and *OeJcCPL2* was significantly decreased compared to WT at four weeks. Compared to WT, *OeJcCPL4* showed a significantly decreased chlorophyll content at eight weeks. The expression levels of several senescence-related genes at eight weeks were also changed. These results indicate that these three *JcCPL* genes function as positive regulators in leaf senescence, especially at the late stage of the plant growth and development. Although there is no literature about CPL genes having such roles, several MYB transcription factors related to the MYB-bHLH-WD40 (MBW) complex have been reported to be involved in the regulation of senescence in plants. GLABRA1 (GL1), a R2R3-MYB transcription factor in *A. thaliana*, is a component of the MBW complex [9,62,63,64]. Besides being involving in trichome formation, *GL1* has a positive role in dark-induced leaf senescence [65]. Several R2R3-MYB genes have also been reported to play roles in anthocyanin accumulation as well as leaf senescence. OsPL is an R2R3-MYB transcription factor, and the mutant *pl* exhibited a higher anthocyanin content and a lower chlorophyll content accompanied by down-regulation of photosynthesis-related genes in flag leaves [66]. In *A. thaliana*, MYB75/PAP1 plays a significant role in plant development mediated by its phosphorylation status, including a delayed onset of senescence when plants overexpress *MYB75*^T131E^ [67]. In *Liquidambar formosana*, *LfMYB113* is a homolog of *A. thaliana MYB75*. Transgenic tobacco containing *35S*::*LfMYB113* shows significant anthocyanin accumulation in leaves, and accelerated leaf senescence [68]. HAT1 interacts with MYB75 and thereby interferes with the MBW protein complex. HAT1 constrains anthocyanin accumulation by inhibiting the activities of this protein complex through blocking the formation of the protein complex and recruiting the TPL corepressor to epigenetically modulate the late anthocyanin biosynthetic genes [69]. Whether the regulation of leaf senescence by the genes *JcCPL1*, *2*, and *4* is related to the MBW complex remains to be elucidated.

## 4. Materials and Methods

### 4.1. Plant Materials and Growth Conditions

The physic nut cultivar GZQX0401 was used in this study. The plants were cultivated in a greenhouse and experimental field at South China Botanical Garden (23°18′ N, 113°36′ E) under natural sunlight [48,70,71].

*Arabidopsis thaliana* (Columbia, Col-0) used for functional analysis of *JcCPL* genes was maintained in a growth cabinet (16 h light/8 h dark, 22 ± 2 °C, 80 µmol m^−2^ s^−1^). For the analysis of anthocyanin content and related genes, plants were grown vertically from sterilized seeds on 1/2 MS medium (1% sucrose and 1% agar, pH 5.8) for 4 days, then transferred to anthocyanin induction medium (AIM) agar plates (1/2 MS, 5% sucrose and 1% agar, pH 5.8) for 7 days. For observation of growth and developmental stage, plants were grown in pots containing a 1:1 mixture of vermiculite and peat moss. Whole shoots were sampled for qRT-PCR and anthocyanin estimation. For senescence-related gene analysis, the fifth and sixth leaves from the apices of 8-week-old plants were sampled and stored at −80 °C.

### 4.2. Sequence Database Searches, Alignment and Phylogenetic Analysis

The amino acid sequences encoded by the CAPRICE (CPC)-like (CPL) genes of *A. thaliana* were used to search for CPLs in the genome and predicted proteome of physic nut by tblastn and blastp, and 6 *JcCPLs* were identified in physic nut. Multiple sequence alignment was performed using Clustal W 1.83 [72] and DNAMAN 6.0 (Lynnon Biosoft Inc., Vandreuil, QC, Canada). The phylogenetic tree was constructed using MEGA 5.0 with neighbor-joining pairwise deletion and bootstrap test 100 times [73].

### 4.3. RNA Isolation and Expression Analysis

Roots, leaves and stems were sampled from 8-week-old physic nut plants. The root samples included all root tips about 10 mm in length, and leaf samples were the fourth fully expanded leaf from the apex, while the stem samples were young bark. Flowers and seeds at points 14, 19, 25, 29, 35, 41 and 45 days after pollination (DAP) were harvested from 5-year-old physic nut plants. All samples were immediately put into liquid nitrogen and stored at −80 °C. The CTAB method was used for isolating total RNA from physic nut organs according to a previous study [70]. The isolation of total RNA from *A. thaliana* shoots and leaves was carried out using Trizol reagent (Invitrogen, Burlington, ON, Canada) following the manufacturer’s instructions.

About 2 μg total RNA (for reverse transcription PCR, RT-PCR) and 5 μg total RNA (for quantitative real-time PCR, qRT-PCR) were used for synthesizing first-strand cDNAs according to the manufacturer’s instructions (Promega, Madison, WI, USA). A *JcActin* (GenBank accession number HM044307.1) gene was used as internal control for physic nut, while *AtACT2* (At3g18780) was used as internal control for *A. thaliana*. Three independent biological replicates, each with three technical repeats, were employed for semi-quantitative RT-PCR and qRT-PCR. For semi-quantitative RT-PCR, PCR products were separated on 1.5% agarose gels and stained with ethidium bromide. The gels were then photographed using a Gel Imaging System (Shanghai Bio-Tech, Shanghai, China), and an LCS480 system (Roche, Rotkreuz, Switzerland) was used for qRT-PCR [71]. The 2^−^^△△CT^ method was used for calculating gene expression levels [74]. The primers used in this work are listed in Supplementary Table S1.

### 4.4. Cloning of JcCPL Genes, Vector Construction and Plant Transformation

The full-length coding sequences of *JcCPL1* (XM_012235954), *JcCPL2* (XM_012236014), *JcCPL3* (XM_020685120), *JcCPL4* (XM_012227851), *JcCPL5* (XM_012230970) and *JcCPL6* (XM_012231357) were amplified by RT-PCR, then they were cloned into the vector pMD18-T (Takara, Shiga, Japan). The primers used in this work are listed in Supplementary Table S1.

For transformation into *A. thaliana*, fragments containing the coding domain sequences (CDS) of the 6 *JcCPLs* were cloned from the pMD18-T vector using the appropriate restriction enzyme sites (*JcCPL1*, *2*, *3*: *Sac* I/*Ps*t I; *JcCPL4*, *6*: *Kpn* I/*Pst* I; *JcCPL5*: *Sac* I/*Sal* I). Then they were ligated into the vector pCAMBIA1301, digested with the same restriction enzyme sites, under the control of the CaMV 35S promoter. The recombinant constructs were transferred into *A. thaliana* plants by the floral-dipping method [75]. Homozygous lines with single T-DNA insertions were selected for subsequent analysis. The primers used in this work are listed in Supplementary Table S1.

### 4.5. Subcellular Localization

For subcellular localization analysis, complete coding sequences without the stop codon were amplified by RT-PCR using the primers listed in Supplementary Table S1. The six modified *JcCPL* cDNAs were cloned into the pSAT6-YFP-N1 vector under the control of the CaMV 35S promoter at the *Eco*R I and *Bam*H I restriction sites.

The naked pSAT6-YFP-N1 vector (YFP) and each of the *JcCPL*-*YFP* recombinants (*JcCPL*-*YFP*) were transformed into *A. thaliana* protoplasts using the PEG-Ca^2+^ method [76]. The plasmid pSAT6-RFP-C1 containing the nuclear localization signal of endonuclease VirD2 under the control of the 35S promoter was co-transformed. For the study, 200 µL protoplast suspension were mixed with 20 µL plasmids (10 µL *YFP*/*JcCPL*-*YFP* + 10 µL *NLS*-*RFP*), and 220 µL PEG-Ca^2+^ solution (40% PEG 4000, 0.2 M mannitol, 0.1 M CaCl_2_,) and incubated at 24 °C for 5 min, then 880 µL solution W5 was added. The mixed suspension was centrifugated at 100× *g* for 2 min, and resuspended in 1 mL W5 solution for 12–24 h at 22 ± 2 °C in the dark. Then the YFP and RFP fluorescence of the protoplasts were observed with a LSM800 laser scanning confocal microscopy system (Carl Zeiss, Oberkochen, Germany). Images were captured at 488 nm and 543 nm laser excitation, and using 493 to 582 nm and 602 to 656 nm long-pass emission filters, for YFP and RFP, respectively.

### 4.6. Measurements of Root Hairs

Seedlings were cultivated on 1/2 MS medium (containing 1% sucrose and 1% agar, pH 5.8) vertically after surface sterilization. After 4 days, seedlings were photographed using a stereo microscope (LEICA, Wetzlar, Germany). Numbers of root hairs within 5 mm in length from the root tips were counted. Thirty seedlings were used for each line.

### 4.7. Measurement of Anthocyanin and Chlorophyll Content

For measurement of anthocyanin content, weighed shoots (ca. 15 mg) were incubated in 1.5 mL hydrochloric acid-methanol mix solution (*v*/*v* = 1:99) at 4 °C for 2 days. The absorbance (A) of the centrifuged extract was determined at 530 nm and 657 nm (A_530_ and A_657_, respectively). The total anthocyanin content was expressed as A_530_ − 0.25A_657_ g^−1^ fresh weight [71,77].

The chlorophyll content was determined using 95% ethanol according to Ding et al. (2018) [78] with minor modification. The contents of chlorophyll a (Chl a), chlorophyll b (Chl b), and total chlorophyll were estimated from leaves (the 3th, 4th, 5th and 6th leaves from the bottom) of transgenic and WT plants at the 3-week stage and 4-week stage, and from leaves (the fifth and sixth leaves from the apex) of transgenic and WT plants at the 8-week stage [79].

### 4.8. Statistical Analysis

At least three biological repeats and technical repeats were used for all experiments, and data were tested for means using SAS software package version 9 with Duncan tests [80].

## Supplementary Materials

The Supplementary Materials are available online at https://www.mdpi.com/article/10.3390/ijms23041924/s1.
