# Supplementary material for: Ectopic Expression of JcCPL1, 2, and 4 Affects Epidermal Cell Differentiation, Anthocyanin Biosynthesis and Leaf Senescence in Arabidopsis thaliana
Source: Int J Mol Sci. 2022 Feb 9;23(4):1924. doi: 10.3390/ijms23041924 (PMC8872631; doi:10.3390/ijms23041924)
Supplement: Supplementary file 1 [file ijms-23-01924-s001.zip › ijms-1549555-supplementary.pdf]

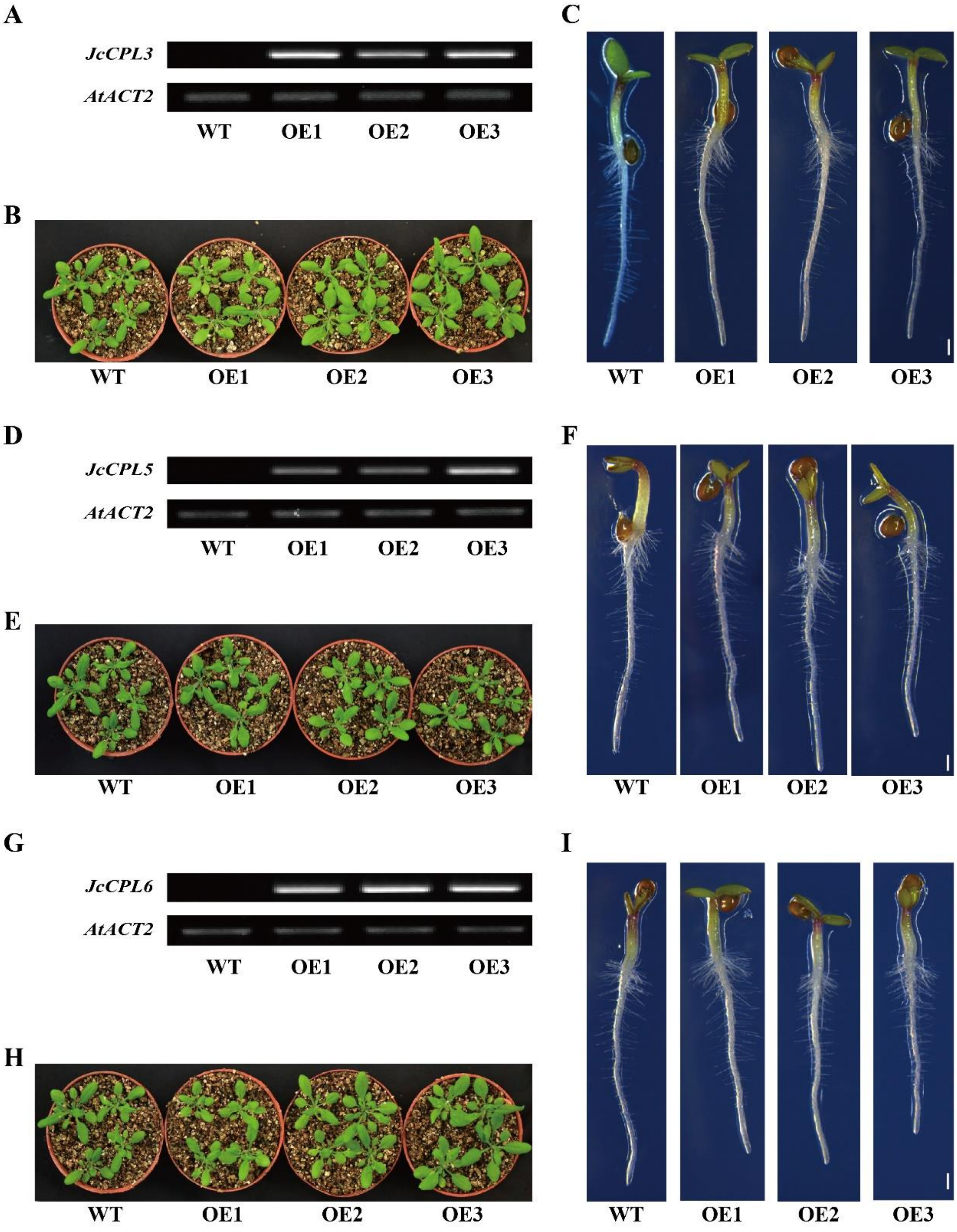

Supplementary Figure S1. Rosette leaves and root hairs of *OeJcCPL3*, *OeJcCPL5* and *OeJcCPL6*. (**A**, **D**, **G**) Expression levels of *JcCPL3* (**A**), *JcCPL5* (**D**) and *JcCPL6* (**G**) in transgenic lines (OE1, OE2 and OE3) determined by semi-quantitative RT-PCR. (**B**, **E**, **H**) Four-week-old seedlings of *OeJcCPL3* (**B**), *OeJcCPL5* (**E**) and *OeJcCPL6* (**H**). (**C**, **F**, **I**) Root hair phenotypes of *OeJcCPL3* (**C**), *OeJcCPL5* (**F**) and *OeJcCPL6* (**I**) grown on 1/2 MS for 3 days. Scale bar = 1 mm.

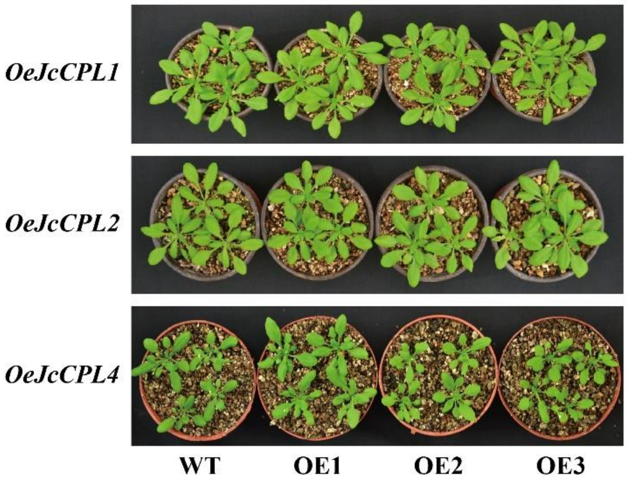

Supplementary Figure S2. Four-week-old seedlings of *OeJcCPL1*, *OeJcCPL2* and *OeJcCPL4*.

Supplementary Table S1. List of primer sequences used in this study.

**A list of primer sequences used in this study (vector construction)**				
**Gene name**	**Plasmids name**	**Restriction enzyme sites**	**Primer sequence （5'-3'）**	
			**Forward**	**Reverse**
JcCPL1	pMD-18T	none	GAAATGGGGGACCAAAATAAC	TTCTGGGTGACTAAACTTCATCTCT
JcCPL2		none	CTCCATTTCTACATAAGTCACATATGGC	CCATTGTCAGCTCCAAAACTTGTAT
JcCPL3		none	GAAAACGATGGACAGACGCA	TTCCGAGTAACAGGGATGGC
JcCPL4		none	AGAAATGGATAGACGCAGAA	TCAGAAGAAGGATGGGATTA
JcCPL5		none	ATTCCTTATGGCTGACTTTG	GCAGCAGCAAGAACCATT
JcCPL6		none	TTATGAAGATGGATGAACGTCC	CCATAGCTAGTAGCTAGGTTGG
JcCPL1	pSAT6--YFP-N1	*EcoRI* / *BamHI*	CCGGAATTCATGGGGGACCAAAATAA	CGGGATCCCTCTCTCAGTTGATGATG
JcCPL2		*EcoRI* / *BamHI*	CCGGAATTCATGGCTGATTCTGAACA	CGGGATCCCTTGACTAGTTGAATATC
JcCPL3		*EcoRI* / *BamHI*	CCGGAATTCATGGACAGACGCAGCCG	CGGGATCCCACATCCAAATTTTATTT
JcCPL4		*EcoRI* / *BamHI*	CCGGAATTCATGGATAGACGCAGAAGG	CGGGATCCCAGACTCAGAGCTCTTGA
JcCPL5		*EcoRI* / *BamHI*	CCGGAATTCATGGCTGACTTTGATC	CGGGATCCCTTGACTTATAGAGTATC
JcCPL6		*EcoRI* / *BamHI*	CCGGAATTCATGGATGAACGTCCATG	CGGGATCCCGTAGCTAGGTTGGTGC
JcCPL1	pCAMBIA1301	*Sac* I / *Pst I*	GCGAGCTCGAAATGGGGGACCAAAATAAC	AACTGCAGTTCTGGGTGACTAAACTTCATCTCT
JcCPL2		*Sac* I / *Pst I*	GCGAGCTCCTCCATTTCTACATAAGTCACATATGGC	AACTGCAGCCATTGTCAGCTCCAAAACTTGTAT
JcCPL3		*Sac* I / *Pst I*	GCGAGCTCGAAAACGATGGACAGACGCA	AACTGCAGTTCCGAGTAACAGGGATGGC
JcCPL4		*Kpn* I / *Pst I*	GGGGTACCAGAAATGGATAGACGCAGAA	AACTGCAGTCAGAAGAAGGATGGGATTA
JcCPL5		*Sac* I / *Sal I*	GCGAGCTCATTCCTTATGGCTGACTTTG	GCGTCGACGCAGCAGCAAGAACCATT
JcCPL6		*Kpn* I / *Pst I*	GGGGTACCTTATGAAGATGGATGAACGTCC	AACTGCAGCCATAGCTAGTAGCTAGGTTGG
